# Wearable Sensors in Other Medical Domains with Application Potential for Orthopedic Trauma Surgery—A Narrative Review

**DOI:** 10.3390/jcm13113134

**Published:** 2024-05-27

**Authors:** Carolina Vogel, Bernd Grimm, Meir T. Marmor, Sureshan Sivananthan, Peter H. Richter, Seth Yarboro, Andrew M. Hanflik, Tina Histing, Benedikt J. Braun

**Affiliations:** 1 University Hospital Tuebingen on Behalf of the Eberhard-Karls-University Tuebingen, BG Unfallklinik, Schnarrenbergstr. 95, 72076 Tuebingen, Germany; cvogel@bgu-tuebingen.de (C.V.); thisting@bgu-tuebingen.de (T.H.); 2 Luxembourg Institute of Health, Department of Precision Health, Human Motion, Orthopaedics, Sports Medicine and Digital Methods Group, 1445 Strassen, Luxembourg; bernd.grimm@lih.lu; 3 Orthopaedic Trauma Institute (OTI), San Francisco General Hospital, University of California, San Francisco, CA 94158, USA; meir.marmor@ucsf.edu; 4 ALTY Orthopaedic Hospital, Kuala Lumpur 50450, Malaysia; drsureshsiva@gmail.com; 5 Department of Trauma and Orthopaedic Surgery, Esslingen Hospotal, 73730 Esslingen, Germany; peter.h.richter@gmail.com; 6 Deptartment Orthopaedic Surgery, University of Virginia, Charlottesville, VA 22908, USA; seth.yarboro@gmail.com; 7 Department of Orthopaedic Surgery, Southern California Permanente Medical Group, Downey Medical Center, Kaiser Permanente, Downey, CA 90027, USA; ahanflik@gmail.com

**Keywords:** orthopedic surgery, digital, body worn sensor, outcome assessment

## Abstract

The use of wearable technology is steadily increasing. In orthopedic trauma surgery, where the musculoskeletal system is directly affected, focus has been directed towards assessing aspects of physical functioning, activity behavior, and mobility/disability. This includes sensors and algorithms to monitor real-world walking speed, daily step counts, ground reaction forces, or range of motion. Several specific reviews have focused on this domain. In other medical fields, wearable sensors and algorithms to monitor digital biometrics have been used with a focus on domain-specific health aspects such as heart rate, sleep, blood oxygen saturation, or fall risk. This review explores the most common clinical and research use cases of wearable sensors in other medical domains and, from it, derives suggestions for the meaningful transfer and application in an orthopedic trauma context.

## 1. Introduction

Digitalization has developed rapidly over the last three decades and has found its way into the private as well as individual health and fitness sector [1,2]. The development of smartphones, smartwatches, and fitness trackers offers the ability to track physical activity and other health-related digital biomarkers [3]. Wearable devices are increasingly being used to complement classic study outcomes to generate additional evidence [2,4]. Depending on their type, wearables contain different arrays of sensors that are positioned directly on the body or in clothing and can measure user and environmental data (i.e., location, meteorological data) continuously over longer time periods [5]. Reviews from the field of orthopedic trauma surgery have already focused on the different applications of these systems within the field [5,6,7,8]. The primary reasons for use are outcome assessment, diagnostics (i.e., event detection of falls), patient engagement, (self-)management, (bio-)feedback, education, rehabilitation/adherence monitoring, and exergaming.

In other medical fields, the research and clinical use of wearable systems have already been established to monitor the treatment and recovery process. These systems are increasingly being used in clinical studies to examine the individual recovery process and to continuously control therapy regimens. Ultimately, this approach enables more personalized, patient-oriented medicine [9,10]. Furthermore, the data extracted from wearables can complement questionnaires, such as Patient-Reported Outcome Measures (PROMs) as an objective correlate [7,11]. In addition, wearable technology can be used as a preventive tool by using biomarkers to provide data to assess the risk of an event (i.e., atrial fibrillation), or minimize risk factors for the onset of a disease [12,13]. While the investigation of digital biomarkers in other fields offers new perspectives for orthopedic trauma surgery, the increasing diversity of systems and limited oversight over tools can be challenging [5,7,8,10,14].

The aim of this review is to provide an overview of the most common clinical and research uses of wearable systems for the diagnostic and outcome/rehabilitation monitoring of patients in other medical domains and to derive suggestions for meaningful transfer into orthopedic trauma surgery practice.

## 2. Cardiology

Basic diagnostics in the field of cardiology include the collection of vital signs such as heart rate, blood pressure (BP), oxygen saturation, and also electrocardiography (ECG). These parameters can be collected through consumer devices, such as smartwatches, in a straightforward and user-friendly manner. It is understandable that the use of wearable technologies is becoming increasingly established in the field of cardiology in research and clinical settings [15,16].

### 2.1. Cardiac Rhythm

Cardiac arrhythmias affect more than 2% of the adult population [17], with atrial fibrillation being most common and increasing the risk of ischemic stroke fivefold [18,19,20]. This increased morbidity and mortality has a corresponding socioeconomic impact, making early detection and timely treatment of this condition desirable [3,21]. Wearables with integrated rhythm detection provide useful data in cardiology diagnostics and tracking, with potential application in trauma. For example, optical photoplethysmography (PPG) sensors can be used to continuously monitor heart rate and thus detect bradycardia or tachycardia [3]. Wearables, such as the Apple Watch or the Fitbit fitness tracker, offer the possibility of detecting potential atrial fibrillation by means of an algorithm based on pulse wave data using PPG [19,20]. In this context, the quality of the data obtained from PPG measurement appears to be close to that of a classic ECG [22]. In addition to the pulse wave data algorithm, an ECG single derivation is also possible with Apple Watches from generation 4. Here, self-diagnosis by users has even been certified by the FDA [3,23]. With the heart rate signal, heart rate variability (HRV) can be assessed as a predictive diagnostic tool for the functional status of the cardiac autonomic nervous system. Recent studies have shown that HRV can be approximately determined by means of a smartwatch under controlled conditions as well as the diagnostic gold standard ECG [24,25].

The analysis of cardiac rhythm and heart rate variability has many potential applications in the field of orthopedic trauma. Preclinical as well as emergency trauma treatment can benefit from wireless measurements of cardiac rhythm and frequency in the setting of both septic and hemorrhagic shock to detect patients at risk for increased morbidity and mortality [26,27,28,29]. While these are certainly settings that have stationary monitoring available for critical patients, those patients deemed non-critical are often transferred out of emergency departments quickly without further cardiac assessment. These patients can benefit from heart rate assessment [27]. This could be easily accomplished with wearable sensors. Furthermore, increased heart rate variability as determined during the early aftercare course can be a predictor of both acute delirium development in geriatric patients, as well as post-traumatic stress disorder in all trauma patients [30,31]. Ultimately, by closely monitoring heart rate during rest, as well as activity, performance diagnostics during rehabilitation can be tracked to assess functional recovery [32].

### 2.2. Blood Pressure

Elevated arterial blood pressure is the most common modifiable cardiovascular risk factor [33]. Wearables that can measure blood pressure without a cuff represent an alternative to classic pneumatic blood pressure monitors [34]. They can estimate blood pressure and thus blood pressure variability, as a prognostic marker for cerebrovascular and cardiovascular disease using a pulse wave analysis algorithm alone or in combination with an ECG [35,36]. Currently, there are inadequate validation protocols for commercial wearables that use PPG and ECG to measure blood pressure [34]. Although user acceptance and feasibility are high, the accuracy of this cuffless wearable blood pressure measurement has yet to be validated in larger-scale studies [37]. An alternative to blood pressure measurement using PPG and ECG is the Watch D smartwatch manufactured by HUAWAEI, which has integrated pneumatic pressure sensors. This smartwatch demonstrated a high accuracy of blood pressure measurement and also has integrated warning systems that notify users of pathological vital signs [38].

Potential short- and long-term applications in trauma include the measurement of patients who lack access to or have compliance issues with stationary or invasive clinical-grade blood pressure monitoring systems. In addition to the more obvious use cases in monitoring patients at risk for low blood pressures in emergency cases, these measurements over a longer time period can help detect patients at risk for inadequate pain control through increased blood pressures, as well as kidney injury due to continued low blood pressure [39,40]. Furthermore, blood pressure can be used as an independent risk factor to assess the risk of prolonged hospitalization, as well as fall risk in geriatric patients, and could be integrated into respective detection workflows [41,42].

### 2.3. Heart Failure

Heart failure is a global health problem with increasing prevalence and high mortality rates, so there is great interest in improving care [43,44,45]. The use of wearable technology allows health data to be obtained beyond the patient’s stay in a medical facility, enabling closer and better patient care. Wearables such as smartwatches or rings can track physical activity and, in combination with applications on the smartphone, prevent the main risk factors of cardiovascular disease, such as obesity and lack of exercise, for example, in digital fitness programs [45]. These preventive programs also have direct implication in primary prevention in trauma patients, as many of the risk factors are known risk factors for problems with wound healing, as well as the development of delayed fracture healing and non-union [46].

Another example of the use of wearables is the TARGET-HF-DM study, which investigates activity levels and also treatment adherence in patients with heart failure and diabetes mellitus. In this study, a pedometer was used as a lifestyle intervention with personalized feedback on physical activity and, through the intervention, was able to produce an increase in the number of daily steps taken and a concomitant improvement in quality of life [47]. This is directly applicable to trauma and has already been proposed as a strategy both in primary as well as secondary prevention [8].

Also, implantables are used in cardiology: In the CHAMPIONS trial, inpatients with heart failure with preserved ejection fraction had a wireless microelectromechanical pulmonary artery pressure monitoring system implanted in the right ventricle as part of a scheduled right heart catheterization procedure, allowing patients to subsequently monitor their own pulmonary artery pressure. The study participants were able to significantly reduce the re-hospitalization rate by 30% within 6 months compared to the control group [48]. While this implantable has few implications for trauma surgery, implantables to measure fracture healing outcome per se will have increasing use in the future, starting with plate-applied strain gages, currently under investigation for market introduction [49].

## 3. Neurology

Neurological diseases are usually accompanied by cognitive and/or motor deficits and thus have a negative impact on quality of life. In this context, wearables such as smartwatches, EEGs, EMGs, and motion sensors can collect continuous, longitudinal data both in the clinical setting and in the home environment and thus influence prevention, diagnostics, and therapy monitoring [50].

### 3.1. Stroke and Neurologic Rehabilitation

With the steadily aging population, the incidence of stroke is increasing [51]. Treatment of these deficits is usually carried out in neurological rehabilitation, which focuses on remobilization and the recovery of daily living skills. In the context of exercise training in stroke patients who walk unassisted, pedometer-guided training can significantly increase physical activity in hospitalized patients [52]. Multiple sequelae due to stroke, such as depression, fatigue, and cognitive impairment, can also be addressed by using physical activity trackers for wearable-based training [53].

Especially in the context of the COVID-19 pandemic, the concept of self-directed rehabilitation in the home environment in combination with telemedicine care has become more established as an alternative to inpatient rehabilitation: The programs focus predominantly on upper-extremity mobility impairments, which are treated using simulation-based training and activity tracking, among other methods. Self-directed rehabilitation is advantageous with regard to exercises in the home environment under real-life conditions [50,54]. In addition to commercially available wearables, sensor systems developed specifically for this purpose, such as gloves with motion sensors, can serve as concrete therapy devices for the convalescence of fine motor skills in neurological rehabilitation [55]. Thus, telerehabilitation with the use of wearables represents a resource-conserving alternative to inpatient rehabilitation, which is associated with higher costs at comparable results [50,55,56].

### 3.2. Neurodegenerative Diseases

Neurodegenerative diseases (NDDs) such as Parkinson’s disease (PD) and Alzheimer’s dementia are typical disorders of aging humans with a high socioeconomic impact, which make effective preventive and therapeutic approaches desirable [57]. The diagnosis of NDDs is mainly based on clinical examination by physicians. Wearable sensors have been investigated for the diagnosis and measurement of disease progression in NDDs [58,59,60]. Moreover, with increasing incidence and a shortage of physician specialists, the treatment of patients with NDDs is becoming increasingly difficult, so the use of telemedicine and wearable devices may provide an alternative perspective to appropriate care [61]. In Parkinson’s disease, for example, smartphone apps can be used to diagnose fine motor impairment in an early stage based on typing characteristics on a touchscreen [62]. A specially developed app accessible on a smartphone can also analyze changes in facial expressions based on a self-portrait photograph, examine emotional status based on text messages, and examine speech and movement patterns [61].

Dementia is a widespread disease that is estimated to affect 82 million people worldwide by 2030 [63]. Despite this global impact, no curative therapy exists to date, so the focus of treatment is on early detection and preserving patients’ functionality and quality of life [64]. Physical inactivity, along with other lifestyle factors, is a relevant risk factor for developing dementia, so promoting physical activity, which has been shown to be increased through the use of wearables such as fitness trackers, is desirable [64,65].

Even in patients with mild cognitive impairment (MCI), increasing physical activity can have a positive impact on disease progression [66]. When examining physical activity, sensor techniques for step counting are currently most commonly used to determine patients’ volume of movement [67]. In this context, objectifying the diagnosis of multifactorial MCI is challenging, as the differences between temporary pathophysiological disease states and an incipient neuropsychiatric disease cannot be reliably differentiated using established questionnaires. The use of wearables, also in combination with smartphone applications, can offer a longitudinal recording of digital biomarkers and cognitive tests, which can improve the classification of MCI and enable adequate therapy [68]. The substantial overlap between patients with MCI/dementia and fracture occurrence makes strategies to prevent movement impairments and falls highly relevant for orthopedic trauma care [69,70].

### 3.3. Sleep

A healthy sleep rhythm is a major determinant of physical health and performance. Sleep deprivation and reduced sleep quality are associated with higher rates of daytime sleepiness and burnout [71]. In highly symptomatic pathologies of sleep, polysomnography in a sleep laboratory is the diagnostic gold standard. The validity of this diagnostic is subject to fluctuations, not least because patients do not sleep in their usual environment and the examination is usually only a one-day snapshot [72]. Wearables with multisensor technology, which enable longitudinal sleep tracking using a wide variety of algorithms, represent a cost-effective alternative [73]. Wearables demonstrate promise in various apps to be able to assess individual sleep using sound recordings and accelerometry data. In some cases, wearables with integrated actigraphy are also coupled with apps that use accelerometers to record data regarding the sleep–wake rhythm: currently, only few validation studies of tracking apps exist, which still calls into question their reliability [73,74,75]. Questions of reliability aside, the impact of sleep for both fracture risk and during recovery is increasingly being recognized, and personal wearable devices can assist in the broad clinical application of sleep research in trauma [76,77,78].

## 4. Geriatrics

The increasing life expectancy of the population is accompanied by an increase in chronic diseases, which cause a reduction in the quality of life and are associated with high socioeconomic costs [79]. The aim of geriatric medicine is the preservation of independence and the avoidance of deterioration in health, which leads to hospital admissions or even the permanent need for care. This also includes the promotion of physical activity, which can have a positive effect on many geriatric diseases [6].

Another challenge is medication adherence among patients discharged from the hospital, estimated to be only 55–70% [80]. On a basic level, apps on smartphones can be programmed to remind patients to take their medications, improving adherence [81,82,83]. This is also true for medication adherence in cardiovascular disease, which represents the leading cause of death worldwide [82,84]. Furthermore, the care of geriatric patients by healthcare professionals is time-consuming and complex, so the use of digital systems to improve care is currently being explored [85,86,87].

### 4.1. Falls

Functional mobility along with cognitive performance are the main factors influencing the risk of a fall. Here, wearables can generate acceleration and rotation data on the lower extremities and provide risk assessments for future falls [88]. Different commercial- and medical-grade sensor types, including accelerometry to plantar pressure, are used to analyze the risk for falling [6,89,90]. More commercial sensors are increasingly being equipped with fall detection capabilities. In combination with digital applications on the smartphone, the fourth-generation Apple Watch, for example, can give fall information and provide new insights into fall prevention. In the GAP Care II study, a specially designed application for the iPhone was successfully used to examine not only vital signs and movement data but also cognitive functions influencing fall risk [91].

### 4.2. Chronic Diseases

Many elderly people suffer from chronic diseases such as diabetes or cardiovascular disease. Wearables, with multifunctional sensor technologies, can provide various opportunities to improve activity and function in elderly patients with chronic diseases as discussed previously. Through the longitudinal collection of digital biomarkers, movement, and environmental information, individual activity can be monitored, allowing for more personalized care that promotes living independently in one’s own home environment for as long as possible [92].

In this context, the use of wearables is one of the gerontotechnological interventions, which also include telemedicine, mobile health applications on smartphones, and remote monitoring systems. Here, wearables provide an interface between the patient and the digital health world [93]. With regard to cardiovascular diseases, wearables such as smartwatches or fitness trackers can digitally record vital signs and send them to healthcare professionals either at regular intervals during medical visits or automatically. However, the use of digital health systems also poses challenges, especially for older patients; the user-friendliness, accessibility, and affordability of technical devices should not be underestimated and may represent a barrier to the widespread use of these technologies [93]. Nonetheless, the use of wearable systems in the United States population is already showing an increasing trend both in the age group of 50 to 64 as well as over 65 years of age with high distribution rates of smartphones and tablets among the aging and elderly [94]

## 5. Discussion

Overall, the distribution of wearable systems is increasing rapidly regardless of medical subspecialty. Decreased mobility and exercise are major risk factors in the development of common medical conditions and injuries and thus affect internal medicine, neurology, geriatrics, as well as orthopedic trauma surgery. Restoring movement and the associated quality of life is an important focus in the care for our patients. The analysis of the multidisciplinary use of wearable technologies opens up new perspectives for orthopedic trauma surgery (Table 1).

### 5.1. Status Quo of Wearable Use in Trauma

Analyzing activity data in trauma patients is an increasing trend to monitor outcome along with PROMs. A review of the literature in orthopedic trauma surgery between 2010 and 2019 has confirmed the increasing presence of these systems in clinical and research settings [8]. This is confirmed by an AO Trauma survey, showing the growing use of wearable systems during clinical treatment [7]. As treatment and recovery in orthopedic trauma surgery are aimed at restoring original function, a new approach utilizing a patient’s own wearable, already used pre-injury, is emerging, called the Bring Your Own Device (BYOD) Approach: by analyzing pre-traumatic activity data, objective baseline parameters for the assessment of the initial physical recovery are obtained, ultimately helping to identify patients at risk for a delayed recovery [8,95,96].

### 5.2. Transfer to Orthopedic Trauma Surgery

Apart from primary prevention, the treatment and monitoring of cardiac and metabolic diseases parallel the immediate, as well as long-term, recovery process after trauma. Patients’ digital biomarkers can provide important parameters postoperatively, such as the longitudinal monitoring of vital signs. In addition, digital self-measurement and possibly automated transmission to hospital information system interfaces could relieve the burden on medical staff and thus save already-scarce resources. After hospital discharge, patients undergoing rehabilitation in order to reduce the physical, psychological, and social consequences of trauma can benefit from wearable-accompanied aftercare [97]. Here, studies on the use of wearables in neurological rehabilitation show alternatives to the self-directed implementation of rehabilitative measures. These are supported by sensor-based activity tracking, exercise guidance through smartphone applications, and telemedicine support for patients [50,55,56]. An example of a concrete rehabilitative intervention is represented by a smartphone application used in PD to examine fine motor skills [62]. A similar smartphone application could be adapted for specific post-treatment regimens in orthopedic traumatology, such as rehabilitation after fractures of the distal radius, similar to guided activity training in lower-extremity fractures. Overall, wearables in orthopedic traumatology rehabilitation could be used to assess functional status pre-injury, as well as during recovery, and guide exercises realistically and under everyday conditions, which may ease acceptability and increase compliance for patients. Furthermore, wearable systems have the potential to increase the activity of users through feedback systems that positively influence treatment outcome [52]. This form of self-directed rehabilitation can strengthen motivation and thus individual self-efficacy, potentially increasing the quality-of-life outcome after injury [53,98].

Especially in elderly patients, who represent a large part of the patient population in orthopedic trauma surgery, falls are a major personal and socioeconomic burden. Wearables are used in geriatrics to collect digital health data in combination with digital cognitive function testing to assess individual fall risk and interact with users through alert systems. Wearables can provide relevant clues to the cause of a fall event based on digital biomarkers such as heart rate, blood pressure, and heart rhythm. Furthermore, wearable-based medication monitoring and adherence can be a transferable application for orthogeriatric trauma treatment. Wearables thus represent digital aids for individual injury prevention and can be a relevant pillar in disease prevention for the steadily aging population.

## 6. A Final Thought

**Figure 1:**
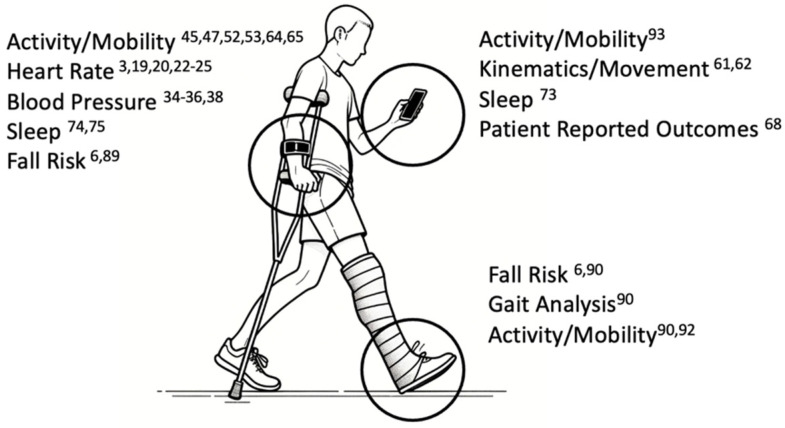
Graphical representation of principal outcome assessed in other fields with application in trauma in relation to the most commonly reported sensor positions (body-worn wearable, left; smartphone, top right; shoe insole, bottom right). Superscript numbers refer to exemplary literature. Graphic created with the help of DALL-E3.

With the advent of digitalization, smart technologies and wearables have become an integral part of many people’s everyday lives. Wearables measure digital biomarkers that we can use in various areas of medicine. In orthopedic trauma surgery, the use of wearables offers unparalleled opportunities in prevention, diagnostics, and outcome assessment during the treatment and rehabilitation process (Figure 1). Here, pre-traumatic, digital activity biopsies can provide an objective impression of the patient’s performance level, vital signs, and movement data and can provide clues from the mechanism of the accident all the way to tracking the individual healing process.

However, looking outside the box with regard to studies reporting on the use of wearables also reveals a need for further research into the methodology, comparability, and general feasibility of studies. Digital biomarkers, including metrics derived from physical activity and function (movement), represent real-life data, such as high-frequency observational data, some of which are incomplete and possibly biased and still need to be subjected to more accurate validation procedures [2]. In addition, studies examining the use of wearable devices have limited applicability to the elderly because they are often underrepresented in the patient population [99]. Future prospective cross-sectional studies need to further investigate the use of wearables in orthopedic trauma surgery with regard to their promising perspectives in individual patient diagnosis and therapy.

More than just identifying use cases with potential application in orthopedic trauma, the analysis of wearable device studies from other fields can also offer learning opportunities concerning the negative aspects of these new technologies and their respective handling. These include challenges associated with wearable device distribution in different patient groups and social demographics [100], the potential negative psychosocial effects of wearable use in patients [101,102], and also the many issues associated with data management, storage, and safety [103,104]. Issues that concern all of us working with these devices both in a clinical as well as research context need addressing. Solutions and adaptations from other fields can be readily applied to trauma and challenges associated with devices, as well as study conduct, resolved with experiences gained in other fields. By combining experiences and efforts across different fields, significant progress concerning aspects of device-agnostic outcome measure standardization and comparability can be expected.

This need for a device-agnostic approach to sensors and algorithms and increased digital biomarker validity across different fields is highlighted by large-scale research projects, such as the Mobilize-D consortium [105]. Currently, a joint qualitative research effort initiated by the AO Foundation linking wearable activity monitor users of different fields is looking to solidify this effort concerning orthopedic trauma surgery. Ultimately, a user-driven network in the form of an online repository listing the most common systems to measure specific outcomes could assist in finding the most appropriate systems for new users while providing a continuous update through expert feedback and ratings.

## Figures and Tables

**Table 1:** Summary of wearable technologies, outcomes, use cases, and potential trauma application.

Specialty and Application	Wearable Technology	Outcome	Potential Trauma Application/Use Case
**Cardiology**			
Heart rate	Optical photoplethysmography (PPG) sensors	Heart rate/heart rate variability Comparable to conventional ECG	Monitoring in emergency and post-emergency settings Heart rate variability to detect patients at risk for increased morbidity/mortality, dementia, and post-traumatic stress disorder Tracking performance recovery during rehabilitation
Blood pressure	Pulse wave analysis ± ECG Pneumatic pressure sensors	Blood pressure	Monitoring in emergency and post-emergency settings Detect patients at risk for inadequate pain control through increased blood pressures Detect patients at risk for kidney injury due to continued low blood pressure Detect geriatric patients at risk for prolonged hospitalization and falls
Heart failure	Accelerometry/pedometer	Step count/activity for personalized training feedback	Rehabilitation tracking during fracture aftercare
**Neurology**			
Stroke and neurologic rehabilitation	Smartphone/accelerometry/pedometer Immersive/non-immersive VR Electrical/vibratory stimulation suits	Activity tracking during rehabilitation Guidance for remote rehabilitation Electrical tissue response/cutaneous mechanoreceptors for rehab	Track rehabilitation progress Enable remote rehabilitation Stimulation of muscle groups after peripheral nerve injury/spinal trauma
Neurodegenerative diseases	Smartphone (screen) Smartphone/wearables (accelerometry)	Detect fine motor impairment through typing characteristics on screen Promotion of physical activity through remote activity assessment	Track rehabilitation of fine motor skills in upper limb injury patients Prevention of inactivity associated negative effects/track rehabilitation progress
Sleep	Smartphone/wearables; multisensor tracking	Sleep time, quality, sleep wake rhythm	Detect patients at risk for falling/fractures through impaired sleep at home Recovery tracking through sleep monitoring in “natural environment” Detect patients at risk for post-OP delirium
**Geriatrics**			
Fall risk	Smartphone/accelerometry/pedometer Plantar pressure	Determine fall risk	Detect patients at risk for falling/fractures in remote settings
Chronic diseases	Multisensor combinations—“Gerotechnology” Smartphone/PPG/blood pressure/accelerometry	Smartphone for monitoring and access to remote health care Smartphone as assistive device to compensate lost functions (i.e., text to speech, voice command) Disease management (i.e., heart rhythm, blood pressure, diabetes) Increasing activity through guided training in obese patients	Application in trauma for screening, fall prevention, and fracture aftercare Overall better remote and digital health access

## Data Availability

No new data were created or analyzed in this study. Data sharing is not applicable to this article.
